# Neural Burst Firing and Its Roles in Mental and Neurological Disorders

**DOI:** 10.3389/fncel.2021.741292

**Published:** 2021-09-27

**Authors:** Jie Shao, Yunhui Liu, Dashuang Gao, Jie Tu, Fan Yang

**Affiliations:** ^1^The Brain Cognition and Brain Disease Institute, Shenzhen Institute of Advanced Technology, Chinese Academy of Sciences, Shenzhen-Hong Kong Institute of Brain Science-Shenzhen Fundamental Research Institutions, Shenzhen, China; ^2^Shenzhen College of Advanced Technology, University of Chinese Academy of Sciences, Beijing, China

**Keywords:** burst firing, ion channels, GPCRs, mental disorders, neurological disorders

## Abstract

Neural firing patterns are critical for specific information coding and transmission, and abnormal firing is implicated in a series of neural pathologies. Recent studies have indicated that enhanced burst firing mediated by T-type voltage-gated calcium channels (T-VGCCs) in specific neuronal subtypes is involved in several mental or neurological disorders such as depression and epilepsy, while suppression of T-VGCCs relieve related symptoms. Burst firing consists of groups of relatively high-frequency spikes separated by quiescence. Neurons in a variety of brain areas, including the thalamus, hypothalamus, cortex, and hippocampus, display burst firing, but the ionic mechanisms that generating burst firing and the related physiological functions vary among regions. In this review, we summarize recent findings on the mechanisms underlying burst firing in various brain areas, as well as the roles of burst firing in several mental and neurological disorders. We also discuss the ion channels and receptors that may regulate burst firing directly or indirectly, with these molecules highlighted as potential intervention targets for the treatment of mental and neurological disorders.

## Introduction

Neurons propagate action potentials with different inter-spike intervals, resulting in various firing patterns such as tonic and burst firing (Gerstner et al., 2014). In several brain regions, neurons can alter their firing pattern from tonic to bursting to regulate emotional states (Yang et al., 2018; Yuan et al., 2019), and abnormal burst firing is implicated in a series of neural pathologies (Sanabria et al., 2001; Fremont et al., 2014; Cain et al., 2018). Neuronal burst firing can enhance neural oscillations (Wang, 1999) and induce neural plasticity (Lisman, 1997; Sieber et al., 2013; Chan et al., 2016). Single burst stimulus given at different phase of θ oscillation can induce long-term potentiation or depression in CA1 *in vitro* (Huerta and Lisman, 1995). In midbrain dopamine (DA) neurons, burst firing stimulus promotes transmitter releasing, and the extracellular dopamine concentration increases in an exponential manner when the stimulation switched from tonic to burst (Gonon, 1988). Bursting promotes DA synaptic releasing but followed by rapid reuptake, which limits its diffusion into extrasynaptic space (Floresco et al., 2003). Moreover, compared with regular stimulation, burst firing evokes greater release of peptides like vasopressin and gonadotropin-releasing hormone (GnRH) in isolated brain tissue (Dutton and Dyball, 1979; Liu et al., 2011). Thus, burst firing appears to be critical in specific message coding and information transmission. Burst firing is not uncommon in the central nervous system (CNS), but its functions and underlying mechanisms vary with brain region and cell subtype. Burst firing in certain areas (especially the thalamus) is initiated by depolarization of low-threshold ion channels (e.g., T-VGCCs) and inward ion channels and is terminated by repolarization (Perez-Reyes, 2003). Thus, burst firing is highly dependent on membrane potential and ion channel dynamics, which offer potential targets for the manipulation of burst firing.

Many ion channels participate in generating or sustaining burst firing, including (but not limited to) voltage-dependent calcium (Cav), sodium (Nav), potassium (Kv) channels, calcium-dependent potassium channels (SK/BK), and hyperpolarization-activated cyclic nucleotide-gated cation (HCN) channels (Zhang et al., 2005; Cueni et al., 2008; Cain et al., 2015; Vandael et al., 2015). Several guanine nucleotide-binding proteins (G protein)-coupled receptors (GPCRs) exert critical influence on neuronal bursting (Wolfe et al., 2003; Tirko et al., 2018). In this review, we discuss the ionic mechanisms and functions of burst firing in several brain areas. We also discuss the potential of intervention in neuronal burst firing via ion channel and receptor modulation as therapeutic treatment for certain neurological disorders.

### Ion Channels Underlying Burst Firing

#### Burst Firing and T-VGCCs

**Figure 1:**
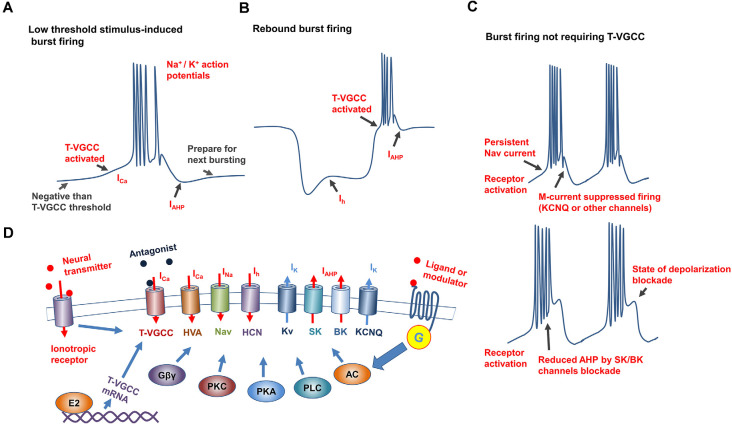
Mechanisms underlying generation and regulation of neuronal burst firing. **(A)** Schematic of burst firing induced by T-VGCC activation caused by slight depolarization, T-VGCCs recover from inactivation by the after hyperpolarization (AHP) to prepare for next bursting. **(B)** Schematic of rebound burst firing, with T-VGCCs activated by hyperpolarization-activated cation current (Ih current) conducted by HCN channels. **(C)** Mechanisms underlying burst firing independent of T-VGCCs. **Top**: Slow inactivation, persistent Nav current induces repetitive action potential firing in hippocampus, M currents [mediated by channels such as Potassium Voltage-Gated Channel Subfamily Q Member (KCNQ)] inhibit membrane excitability, and participate in regulating of frequency and duration of burst firing. **Bottom**: Putative mechanism underlying burst firing of substantia nigra dopamine neurons induced by SK/BK channel inhibition *in vitro*. After excitation, when calcium-activated potassium channels are inhibited, membrane gradually reaches a state of depolarization blockade. **(D)** Mechanisms underlying burst firing regulation. Conductance of ion channels underlying burst firing is affected by membrane potential, antagonists, and G-protein-coupled (GPCR) and ionotropic receptor activity. Altered expression by E2 of T-VGCCs affects T-type currents, which directly regulates burst firing.

Among the ionic mechanisms underlying burst firing, the low-threshold response induced by T-VGCCs is a common cellular mechanism for initiating bursting in several brain regions(Cain and Snutch, 2010). The T-VGCC family consists of three widely expressed subtypes: Cav 3.1, Cav 3.2, and Cav 3.3 (Perez-Reyes, 2003; Cain and Snutch, 2011). Compared with other Cav channels, T-VGCC can be activated at more hyperpolarized membrane potentials (between −70 and −60 mV) with slower inactivation kinetics (McRory et al., 2001; Perez-Reyes, 2003). These biophysical properties enable T-VGCC-expressing neurons to be depolarized at low threshold (initial membrane potential should be hyperpolarized to ensure T-VGCC activation), and thus reach the threshold of Nav channels to fire a group of spikes over a short period before inactivation (Figure 1A). Repolarization channels, such as Kv and SK channels, are activated to rapidly hyperpolarize neurons back to their resting membrane potential, allowing T-VGCCs to recover from an inactive state and prepare for the next burst (Perez-Reyes, 2003). In this case, T-VGCC mediated bursting will produce pacemaker activity, which can affect neuronal oscillations. Despite their role in generating synchronous activity, T-VGCCs are also implicated in modulating synaptic connections. Low-threshold calcium spikes in the dendrites of thalamocortical relay neurons are essential for inhibitory postsynaptic long-term potentiation (Sieber et al., 2013). T-VGCCs in the dendrites of hippocampal CA1 neurons enable subthreshold synaptic potential to induce local depolarizing potential (Magee et al., 1995).

Many antagonists of T-VGCCs exert blockade of all three subtypes, and include nickel, mibefradil, and Z944 (Zamponi et al., 1996; Marks et al., 2016b; Yang et al., 2018). Considering subtype specificity, isoform blockade by nickel displays regional, tissue-dependent variability, and Cav3.2 show the highest sensitivity to nickel among isoforms (Zamponi et al., 1996; Kang et al., 2006). Extracellular histidine residue of glutamine at position 191 of Cav 3.2 is a high-affinity nickel binding site, and ascorbate acts through this site to inhibit the inward calcium current by activating Cav 3.2 (Nelson et al., 2007). High antagonist sensitivity makes it easier to block Cav 3.2, but it is much more difficult to discriminate Cav3.1 and Cav3.3 from other isoforms. As such, greater effort should be expended on developing and designing drugs that specifically modulate each isoform. Lack of subtype-specific antagonists makes it difficult to determine the differences in the properties and functions of the isoforms. However, transient expression of the three subtypes in cultured cells indicates that the Cav3.3 isoform is activated and inactivated more slowly than the other two isoforms but at the most hyperpolarized thresholds. Furthermore, the Cav3.1 subtype exhibits the fastest activation and inactivation kinetics (Kozlov et al., 1999; McRory et al., 2001). In thalamic relay neurons displaying rebound-bursting, replacing T-VGCCs with Cav3.1 results in decreased firing rates, whereas substitution of Cav3.3 has no obvious effect on burst firing and Cav3.2 only produces one rebound spike (McRory et al., 2001). This suggests that the T-VGCC isoforms possess distinct electrophysiological properties and different contributions to the generation of bursts.

In addition to the distinct characteristics of the three isoforms, the properties of T-VGCC-mediated burst firing depend on the neuronal cell type in the different brain areas. Calcium currents conducted by T-VGCCs in GnRH neurons exhibit relatively slow inactivation kinetics, indicating that Cav3.3 may be the most important subtype for burst firing in these neurons (Zhang et al., 2009). In the thalamus, Cav3.1 is expressed in the ventrobasal and laterodorsal thalamic neurons, Cav3.3 is expressed in the laterodorsal and reticular parts, and Cav3.2 is expressed in the reticular thalamus only (Nelson et al., 2007). Cav3.2 knockout in dentate gyrus neurons impairs the ability to fire in a burst pattern (Dumenieu et al., 2018). Certain subpopulations in the medial habenula (MHB) exhibit burst firing, relying primarily on the function of Cav3.1 (Vickstrom et al., 2020). The fast kinetics of low threshold currents suggest the possible contribution of Cav3.1 to bursting in the subthalamic nucleus (Tai et al., 2011). Together, the variability in T-type currents in different brain regions may reflect differential expression patterns of Cav3 channel isoforms, which lead to regional specificity in T-VGCC-induced burst firing.

T-VGCC expression is also highly related to burst firing. Previous research has shown that T-VGCC expression and burst firing in the ventral subiculum of stress-susceptible mice are enhanced after long periods of defeat-stress by aggressor mice, although the underlying mechanism remains unclear (Lee et al., 2019b). 17β-Estradiol (E2) can modulate T-VGCC expression with subtype- and brain region-specificity through the estrogen receptor (Bosch et al., 2009). Following E2 administration, T-VGCCs are up-regulated in several hypothalamic nuclei, resulting in enhanced T-type calcium currents and rebound bursting. In the pituitary gland, Cav3.1 expression is increased by E2 application, and T-VGCC-mediated burst firing in the pituitary gland is considered to modulate hormone secretion (Qiu et al., 2006). E2 treatment also up-regulates the expression of the three T-VGCC subtypes in GnRH neurons, which, in turn, increases the T-type calcium current to promote burst firing (Zhang et al., 2009). Moreover, alternate splicing events during T-VGCC expression can result in changes in the T-type current, which may affect burst firing (Zhong et al., 2006). In summary, T-VGCC conductance and expression can be manipulated to regulate the function of burst firing.

#### Burst Firing and Other Ion Channels

Other channels, including high threshold Cav channels, persistent Nav channels, SK/BK channels, and HCN channels, also modulate membrane potential to sustain burst firing or directly initiate burst firing (Carr et al., 2003; Zhang et al., 2005; Rouchet et al., 2008; Cain et al., 2015; Niday and Bean, 2021). However, these induced burst firings display different properties compared with T-VGCC-mediated bursting. Previous studies have reported that DA neurons in several areas display high-frequency bursts, although the underling mechanism is complex (Grace and Bunney, 1984; Zhang et al., 2005; Blythe et al., 2009): in the substantia nigra (SN), activation of Cav1 channels increases burst firing by inhibiting K^+^ currents ; blocked of high voltage-gated Cav1.2 and Cav1.3 in the ventral tegmental area (VTA) significantly suppresses the bursting firing, while enhanced calcium influx conducted by these L-type (not T-type) channels induces burst firing directly. Many Kv channels also contribute to the regulation of burst firing, and their activation can hyperpolarize membranes and re-activate T-VGCCs (Perez-Reyes, 2003). The slow inactivation kinetics of persistent Nav channels enables the shaping of repetitive firing in the hippocampus (Carr et al., 2003). Activation of oxytocin receptors can induce burst firing in the hippocampal CA2 area, with the duration and spiking rate of bursting modulated by the dynamics of the KCNQ (Potassium Voltage-Gated Channel Subfamily Q Member) and persistent Nav channels (Tirko et al., 2018). Furthermore, activation of metabotropic glutamate receptor depolarizes SN DA neuronal membranes and induces firing, as well as reduced activity of small conductance SK channels, can shift this regular firing into bursting *in vitro* (Prisco et al., 2002). Similar effects of SK channel blockade on burst firing of CA2 neurons have been observed in some serotoninergic neurons in the dorsal raphe (Rouchet et al., 2008). Inhibiting large conductance (BK) calcium-activated potassium channel currents in acutely isolated Purkinje neurons can convert the tonic firing of single action potentials into burst firing (Figure 1C; Niday and Bean, 2021). However, burst firings induced by SK/BK channel inhibition have mainly been performed mainly *in vitro* with pharmacological tools, and whether these channels facilitate burst firing *in vivo* requires more evidence.

The HCN channel is an important pacemaker and modulator of cardiac and neuronal excitability, and contributes to bursting in many neurons, especially rebound burst firing (Figure 1B; Cain et al., 2015). In genetic absence epilepsy rats from the Strasbourg (GAERS) model, ventrobasal thalamic neurons display an inherent suppression of burst firing, concomitant with an enhanced basal Ih current (Cain et al., 2015). Hyperpolarization of membrane potential activates HCN channels to produce Ih currents and slightly depolarize the membrane. Due to the nature of HCN channels, the enhancement of Ih currents in GAERS ventrobasal thalamic neurons inhibits hyperpolarization, and thus they require a significantly larger hyperpolarized current injection to de-inactivate T-VGCCs and produce rebound burst firing (Cain et al., 2015). Consistent with this, a decrease in the HCN1 channel is reported to occur in cortical neuron dendrites in the GAERS model, which contributes to calcium electrogenesis and neuronal bursting(Kole et al., 2007). However, ZD7288 treatment (antagonist of HCN channel) exerts an obvious inhibitory effect on burst firing in lateral habenula (LHB) neurons(Yang et al., 2018). Although the mechanism underlying HCN regulation of bursting is complex, interactions with the T-type calcium window and SK channel currents are considered to dominantly affect burst firing.

### GPCRs Involved in Regulation of Burst Firing

Burst firing is tightly related to the activation and inactivation kinetics of ion channels like T-VGCCs (Nelson et al., 2007). In the CNS, GPCRs [such as metabotropic glutamate receptors (mGluRs)] bind with neurotransmitters or neuromodulators to activate downstream protein kinases, and thereby phosphorylate various ion channels and alter neuronal electrophysiological properties (Reiner and Levitz, 2018). These properties hint at the potential mechanism underlying the regulation of burst firing by GPCRs. Indeed, many studies have reported that neurotransmitters and neuromodulators can alter neuronal firing properties (Cantrell et al., 1996; Tirko et al., 2018) and transform firing pattern from tonic to bursting. Here, we explore the involvement of GPCRs in regulating burst firing; and discuss the possible treatment of burst firing-induced malfunctions by GPCR function manipulation.

#### GPCRs Regulate T-VGCC-Mediated Burst Firing

GPCRs can directly modulate T-VGCCs to regulate burst firing in many cell subtypes. Activation of mGluRs in cerebellar Purkinje neurons promotes T-VGCC currents (mainly Cav3.1), which rely on tyrosine phosphatase/kinase to enhance Cav3.1 conductance (Hildebrand et al., 2009). However, blockade of the mGluR1-phospholipase C beta4 (PLC beta4) pathway in thalamocortical neurons promotes T-VGCC currents and thus enhances burst firing (Cheong et al., 2008). Activation of corticotropin-releasing factor receptor 1 inhibits Cav3.2 through G protein βγ subunits (Tao et al., 2008), mainly via binding to intracellular loop linking transmembrane domains (Wolfe et al., 2003). Serotonin (5-HT_7_) receptors can affect adenylyl cyclase via the G_sα_ protein. In rats, 5-HT application in the adrenal glands activate 5-HT_7_ receptors and enhances adenylyl cyclase activity, which increases T-type calcium currents (Lenglet et al., 2002). However, activation of the 5-HT_2_ receptors in olivary neurons is reported to inhibit calcium influx through T-VGCCs (Placantonakis et al., 2000), with the same inhibition observed in T-VGCC-induced burst firing in subiculum neurons after activation of 5-HT_2C_ receptors (Petersen et al., 2017). The 5-HT_2C_ receptors belong to GPCRs coupled to G_αq_ and G_βγ_. Activation of G_αq_-coupled neurokinin 1 receptor and related protein kinase C (PKC) and phospholipase C (PLC) inhibits Cav3.2 channels in cultured cells(Rangel et al., 2010), and G_βγ_ subunits also suppress Cav3.2 conductance, as mentioned above. Taken together, this evidence indicates that activation of GPCRs affects the dynamics of T-VGCCs with neuronal subtype specificity.

#### GPCRs Regulate Persistent Nav Current-Mediated Burst Firing

Persistent Nav currents induce repetitive burst firing in many brain regions (Figure 1C), especially the hippocampus (Azouz et al., 1996; Carr et al., 2003). The hippocampus receives abundant cholinergic signals from the upstream basal forebrain (Frotscher and Léránth, 1985), and activity of muscarinic receptors is associated with the firing mode of the hippocampus neuronal subpopulations (Benardo and Prince, 1982). Muscarinic receptors belong to GPCRs (Bonner et al., 1987), and activation of muscarinic receptors with different concentrations of agonists exerts complicated effects on neuronal firing patterns (Benardo and Prince, 1982; Alroy et al., 1999). Application of low-concentration agonists to muscarinic receptors releases second messengers and activates PKC(Caulfield, 1993), which inhibits persistent Nav currents and related burst firing. In this scenario, the low concentrations of muscarinic receptor agonists switch the firing patterns of hippocampal CA1 neurons from bursting to consecutive tonic firing (Azouz et al., 1994; Alroy et al., 1999).

In contrast, several hippocampal-based studies have shown enhancement of bursting activity by muscarinic receptor activation (Benardo and Prince, 1982; Kawasaki and Avoli, 1996). These enhanced effects may be due to the PKC-dependent suppression of repolarized K^+^ currents induced by the application of muscarinic agonists at high concentration, thereby promoting inward Ca^2+^ currents and burst firing (Brown et al., 1997). The activation of voltage-dependent cationic non-selective currents by muscarinic agonists may be another potential mechanism (Haj-Dahmane and Andrade, 1996). However, the concentration of agonist used in the above studies may exceed the physiological concentration of acetylcholine released by cholinergic terminals, and the enhanced effects are mainly induced by affecting membrane potential. Thus, burst firing mediated by persistent Nav currents in hippocampus may be inhibited by cholinergic signals under physiological conditions.

Oxytocin is reported to play a critical role in modulating social behavior (Jones et al., 2017; Anpilov et al., 2020), with most effects initiated through the oxytocin receptor (GPCR binding with G_αi/q_ proteins; Jurek and Neumann, 2018). In the hippocampal CA2 area, activation of oxytocin receptors by specific agonist [Thr4, Gly7]-oxytocin (TGOT) can induce pyramidal neuronal firing in bursting pattern. Persistent Nav channels are involved in the generation of burst firing and regulated by TGOT-induced PKC activation. Furthermore, inhibition of KCNQ by TGOT provides sustained depolarization to drive burst firing (Tirko et al., 2018).

Collectively, the above studies demonstrate that GPCRs exert brain region- and cell subtype-specific effects on T-VGCCs and associated ion channels to modulate burst firing (Figure 1D). However, exploration of GPCRs as potential targets to intervene in abnormal burst firing is still lacking. Due to the complicated relationship between GPCRs and burst firing, future research should focus on the possibility of manipulating specific GPCRs to rescue burst firing-induced malfunctions.

### Function of Burst Firing in Specific Brain Regions and Its Role in Neurological Disease

#### Physiological Functions of Burst Firing in Specific Brain Regions

Under normal physiological conditions, burst firing can be found in various brain areas, such as the hypothalamus, thalamus, hippocampus, and cortex; however, its functions vary in different areas and cell subtypes. In the hypothalamus, GnRH neurons display T-VGCC-dependent burst firing, which can promote a GnRH and luteinizing hormone surge (Zhang et al., 2009). The ventromedial nucleus of the hypothalamus (VMH) shows high expression of T-VGCCs (Qiu et al., 2006), and oscillation activity in the VMH is tightly correlated with oscillation of sympathetic nerve activity (Iigaya et al., 2017). Superfusion with anorexigenic peptides, such as insulin, increases the spiking rate of VMH oscillations, while application of orexigenic peptides like leptin decreases oscillation frequency (Iigaya et al., 2019). These metabolism-related peptides may affect oscillation via depolarizing/hyperpolarizing the neuronal membrane, but the effect of altered oscillation on the sympathetic system is unignorable. These findings indicate that burst firing may be important in the hypothalamic regulation of secretion and metabolism.

In the thalamus, neuronal firing patterns are highly related to distinct sensory messages propagated to the cortex (Cheong et al., 2008). The pacemaker activity of the thalamic reticular nucleus induced by T-VGCC-mediated burst firing contributes to the appearance of oscillating spindles during non-rapid eye movement (Halassa et al., 2011). In thalamocortical neurons, increased bursting and decreased tonic firing reduce visceral pain responses (Cheong et al., 2008), contrary to the function of burst firing in dorsal root ganglia sensory neurons, which amplify peripheral pain signals (Jagodic et al., 2007).

In the hippocampus, the subiculum receives outputs from the hippocampal CA1 and thus regulates memory formation and spatial information processing (Joksimovic et al., 2017). T-VGCCs, especially Cav3.1, are abundantly expressed in subiculum neurons, which contributes to the generation of neuronal burst firing. Neurons in the subiculum that bursting infrequently exhibit stronger spatial modulation than those neurons that predominantly show burst firing (Simonnet and Brecht, 2019). Activation of oxytocin receptors in CA2 neurons enhances the excitatory-inhibitory ratio and plasticity of synapses connected to CA1 neurons, which are crucial for social memory processing and spatial representation (Tirko et al., 2018).

As the release of DA is associated with spiking rate, burst firing of midbrain DA neurons (including the SN and ventral tegmental area) in response to reward-predictive stimuli or reward better than expected significantly increases terminal DA phasic release in downstream targets like the nucleus accumbens and prefrontal cortex, and therefore promotes the motivation of reward-seeking and reward-driven learning (Schultz, 1998, 2002; Floresco et al., 2003). In this case, burst firing of DA neurons may code this prediction error and reinforces learning by modifying DA synaptic transmission. Burst firing and burst synchronization in the prefrontal and anterior cingulate cortices promote attention-focusing (Womelsdorf et al., 2014). Together, these studies highlight the critical role of burst firing in the maintenance of normal function of various neuronal subtypes.

### Burst Firing and Related Diseases in CNS

Increasing evidence indicates that certain neuronal subpopulations display abnormal burst firing or shift firing patterns from tonic to burst firing in several nuclei during pathophysiological progress of certain neurological diseases.

*Anxiety* is an emotion state defined as the anticipation of future or potential threat and stress. Normal adaptive anxiety can promote individual survival, however, excessive pathological anxiety will have detrimental effects on the mind and body and requires clinical treatment (Crocq, 2015). Abnormal burst firing is reportedly involved in the pathophysiological processes of anxiety. In the hypothalamic paraventricular nucleus, exposure to chronic stress promotes neuronal burst firing and anxiety-like behavior, while reward prevents these effects (Yuan et al., 2019). Short bursts of spiking in dorsal raphe serotoninergic neurons enhances the release of serotonin (Gartside et al., 2000), which is critical for the regulation of anxiety state (Hornung, 2003). T-type calcium currents regulate excitability of medial amygdala neurons by inducing burst firing, and bursting in these neurons is significantly enhanced in rats under an anxiety state induced by spinal nerve ligation (Jiang et al., 2014; Padilla et al., 2016). The VMH is suggested to regulate anxiety-like behavior (Kunwar et al., 2015; Cho et al., 2020). However, whether T-VGCCs, which are located in the VMH (Qiu et al., 2006) mediate burst firing in the VMH and regulate anxiety requires further investigation.

*Depression* is a mood disorder characterized by loss of interest in activity, accompanied by increased risk of suicidal behavior and psychiatric and psychosocial morbidity (Birmaher et al., 2002). The LHB is suggested to be an important node in regulating depression and related aversive behavior (Li et al., 2013). In animal models of depression, LHB neurons display increased burst firing, which is predicted to promote transmitter release to downstream brain regions (Yang et al., 2018).

LHB burst firing relies on T-VGCC dynamics (Yang et al., 2018). Based on the nature of T-VGCCs, LHB burst firing could potentially be manipulated through hyperpolarizing or depolarizing membrane potentials. Optogenetic hyperpolarization of LHB neurons can induce rebound burst firing, while knockdown of astroglia Kir4.1 can slightly depolarize the LHB neuronal membrane to suppress T-VGCCs (Cui et al., 2018). In addition, increased T-VGCC expression in the ventral subiculum can occur after long-term social defeat (especially in the nucleus accumbens-projecting subpopulation), and enhanced T-VGCC-mediated bursting may contribute to the development of depression (Lee et al., 2019a, b). These studies suggest a close link between depression and T-VGCC-mediated burst firing, and blockade of T-VGCC or suppression of T-VGCC-mediated burst firing could be a potential therapy for depression.

*Epilepsy* refers to several neurological disorders characterized by recurrent seizure, with excessive excitation or hypersynchronous neural activity (Fisher et al., 2014). Many factors may contribute to excessive and synchronous neural firing during epileptic seizure, such as reduced inhibitory activity or abnormal function of certain ion channels (Rowley et al., 2012; Wei et al., 2017). T-VGCC activation produces a low-threshold calcium potential, which initiates a depolarization cascade of neurons and generates high-frequency burst firing (Joksimovic et al., 2017). This burst firing is predicted to generate the hyper-synchronization of neurons and spread oscillations which promote absence seizure (Cain et al., 2018). The three T-VGCC subtypes are widely expressed in thalamic and thalamocortical neurons with subtype-specificity (Khosravani et al., 2004; Cain et al., 2018). Previous studies have suggested that T-VGCCs blocked in the thalamus suppress the neuronal synchronous firing that underlies absence seizures (Tringham et al., 2012). Activation of the thalamic reticular nucleus *in vivo* promotes the rebound burst firing in thalamocortical relay neurons (Halassa et al., 2011), this T-VGCC-mediated burst firing is critical for the genesis of synchronous spike-and-wave discharges during absence seizure (Kim et al., 2001). Together, T-VGCC-mediated bursting in these neurons is tightly related to the genesis of absence seizure.

Based on the relationship between T-VGCCs and absence seizure, antagonists of T-VGCCs are considered as potential anti-epileptic drugs. Long-term oral application of ethosuximide suppresses seizures in many rat models of absence epilepsy, including Wistar albino Glaxo/Rijswijk (WAG/Rij) and GAERS model rats, and its anti-epileptic effects mainly act via inhibition of T-VGCCs (Blumenfeld et al., 2008; Dezsi et al., 2013). In addition, application of T-VGCC antagonist Z944 rescues impaired visual recognition memory in the GAERS model (Marks et al., 2016a). These studies suggest the potential use of selective antagonists of T-VGCCs in the clinical intervention of absence epilepsy and related syndromes. However, although certain evidence indicates that T-VGCCs in the hippocampus may contribute to the epileptogenesis of temporal lobe epilepsy (TLE), which is caused by abnormal structural or metabolic conditions (Sanabria et al., 2001; Becker et al., 2008), whether T-VGCC antagonists can effectively ameliorate TLE require further study.

*Parkinson’s disease (PD)* is a progressive neurodegenerative disease manifested with core motor symptoms like tremor. Oscillation due to central neuronal pacemakers is considered an important mechanism underlying rhythmic oscillatory activity in such tremors (Deuschl et al., 2001). Specifically, burst firing in inferior olivary neurons can transmit through the cerebellum and thus exert an important influence on the control of movement, with this oscillatory behavior determined by T-VGCC expression (Handforth et al., 2010; Bazzigaluppi and de Jeu, 2016). Administration of the T-VGCC antagonist zonisamide in PD animal models suppresses tremulous jaw movements which are postulated to affect olivocerebellar system rhythmicity (Miwa et al., 2011). Furthermore, enhanced neuronal burst firing in the STN is implicated in the pathology of PD, and application of T-VGCC antagonists rescues motor disabilities in PD rats induced by 6-hydroxydopamine injection (Tai et al., 2011). Moreover, neurons in deep cerebellar nuclei exhibit bursting activity during rapid-onset dystonia parkinsonism, while Nav channel inhibition alleviates this dystonia (Fremont et al., 2014). Blockade of Cav1 channels partially rescues SN DA neurons in PD by inhibiting burst firing (Blythe et al., 2009). The above studies demonstrate that burst firing manipulation may be a possible treatment for PD tremor.

*Other diseases* are also related to neuronal burst firing. Several mental disorders, such as schizophrenia and addiction are tightly associated with abnormal neural activity of the DA neurons, while cholinergic input to DA neurons exerts an important influence on their firing pattern (Schultz, 2002; Zhang et al., 2005); however, the direct connection between burst firing and mental disorders like schizophrenia needs further study. In animal models of addiction, burst firing of VTA DA neurons alters transmitter release into reward-regulating downstream regions such as nucleus accumbens (Schultz, 2002; Floresco et al., 2003). However, the link between addiction and burst firing remains elusive. Moreover, the social behavior impairment of schizophrenia (Penn et al., 2008) is thought to be related to CA2 neuronal reduction (Benes et al., 2008). As such, manipulation of burst firing through oxytocin receptors in CA2 may facilitate neural circuits to restore the malfunctions of social behavior manifested by schizophrenia and other diseases (Wang, 1999). Genetic mutation of Cav3.2 is reported to result in a reduction in T-type currents, which may be involved in autism spectrum disorder (Splawski et al., 2006). In conclusion, these studies suggest the important role of neuronal burst firing in the pathology of many neurological diseases. However, further research is required to test the feasibility of antagonist and GPCR application in modulating burst firing for the treatment of such diseases.

## Conclusions

Burst firing is a common pattern in the CNS, however the ionic mechanism of burst firing varies in different neuronal subtypes. T-VGCC activation-induced low-threshold spiking is the most common and important mechanism initiating neuronal burst firing, but its properties vary depending on the differentially expressed isoforms of T-VGCCs. Other channels, e.g., persistent Nav and KCNQ channels, also contribute to burst firing generation and maintenance in several neuronal subtypes. Burst firing in different neuronal subtypes displays distinct functions, and abnormal burst firing caused by changes in T-VGCC conductance (or that of other channels) is thought to be involved in the pathology of several neurological and physical diseases, including anxiety, depression, and epilepsy. Importantly, antagonists and GPCRs affecting those ion channels can regulate neuronal burst firing, suggesting potential application for the clinical treatment of anxiety, depression, epilepsy, and other burst firing-induced syndromes. Moreover, neurons in many brain regions exhibit burst firing (Qiu et al., 2006) but lack detailed exploration. Thus, clarifying the specific functions and underlying mechanisms of burst firing in the CNS could facilitate treatment of these diseases.

## Author Contributions

JS and YL wrote the draft of the manuscript. FY designed, wrote, checked, and finalized the manuscript. All authors contributed to the article and approved the submitted version.

## Conflict of Interest

The authors declare that the research was conducted in the absence of any commercial or financial relationships that could be construed as a potential conflict of interest.

## Publisher’s Note

All claims expressed in this article are solely those of the authors and do not necessarily represent those of their affiliated organizations, or those of the publisher, the editors and the reviewers. Any product that may be evaluated in this article, or claim that may be made by its manufacturer, is not guaranteed or endorsed by the publisher.
